# Value and Quality of Care in Head and Neck Oncology

**DOI:** 10.1007/s11912-020-00952-5

**Published:** 2020-07-10

**Authors:** Robert P. Takes, Gyorgy B. Halmos, John A. Ridge, Paolo Bossi, Matthias A.W. Merkx, Alessandra Rinaldo, Alvaro Sanabria, Ludi E. Smeele, Antti A. Mäkitie, Alfio Ferlito

**Affiliations:** 1 Department of Otolaryngology/Head and Neck Surgery, Radboud University Medical Center, P.O. Box 9101, 6500 HB Nijmegen, The Netherlands; 2 Department of Otorhinolaryngology - Head and Neck Surgery, University Medical Center Groningen, University of Groningen, Groningen, The Netherlands; 3 Department of Surgical Oncology, Fox Chase Cancer Center, Philadelphia, PA USA; 4 Department of Medical and Surgical Specialties, Radiological Sciences and Public Health, University of Brescia, ASST Spedali Civili, Brescia, Italy; 5 Department of Oral and Maxillofacial Surgery, Radboud University Medical Centre, Nijmegen, The Netherlands; 6 University of Udine School of Medicine, Udine, Italy; 7 Department of Surgery, School of Medicine, Hospital Universitario San Vicente Fundacion. CEXCA Centro de Excelencia en Enfermedades de Cabeza y Cuello, Universidad de Antioquia, Medellín, Colombia; 8 Department of Head and Neck Oncology and Surgery, Antoni Van Leeuwenhoek Hospital, Netherlands Cancer Institute, Plesmanlaan 121, 1066 CX Amsterdam, The Netherlands; 9 Department of Oral and Maxillofacial Surgery, Amsterdam UMC, University of Amsterdam, Amsterdam, Netherlands; 10 Department of Otorhinolaryngology - Head and Neck Surgery, University of Helsinki and HUS Helsinki University Hospital, Helsinki, Finland; 11 International Head and Neck Scientific Group, Padua, Italy

**Keywords:** Head and neck, Cancer, Quality of care, Indicators, Value-based health care

## Abstract

**Purpose of Review:**

The concept of value-based health care (VBHC) was articulated more than a decade ago. However, its clinical implementation remains an on-going process and a particularly demanding one for the domain of head and neck cancer (HNC). These cancers often present with fast growing tumors in functionally and cosmetically sensitive sites and afflict patients with differing circumstances and comorbidity. Moreover, the various treatment modalities and protocols have different effects on functional outcomes. Hence, the interpretation of what constitutes VBHC in head and neck oncology remains challenging.

**Recent Findings:**

This monograph reviews developments in specific aspects of VBHC for HNC patients, including establishment of registries and quality indices (such as infrastructure, process, and outcome indicators). It emphasizes the importance of the multidisciplinary team, “time to treatment intervals,” and adherence to guidelines. The discussion addresses major indicators including survival, quality of life and functional outcomes, and adverse events. Also, strengths and weaknesses of nomograms, prognostic and decision models, and variation of care warrant attention.

**Summary:**

Health care professionals, together with patients, must properly define quality and relevant outcomes, both for the individual patient as well as the HNC population. It is essential to capture and organize the relevant data so that they can be analyzed and the results used to improve both outcomes and value.

## Introduction

Head and neck cancer (HNC) is characterized by often relatively fast growing tumors in anatomically delicate and functionally vulnerable sites. Tumor progression, resection, and sequelae of non-surgical management may all eventuate in durable functional decline. Whereas survival has long been acknowledged as an important outcome of HNC care, quality of life is increasingly recognized. Pursuit of cure by intensifying treatment is usually associated with increased adverse events (such acute toxicity and postoperative complications) as well as long-term morbidity. This may be acceptable when balanced against the survival benefits or better quality of life. Therefore, when defining benefits or outcomes of HNC treatment, both oncologic results as well as quality of life are important.

With increasing costs of care in many Western societies, sustaining their health care systems creates problems that cannot be ignored. Thus, recent years have brought an increased focus on assessing value (i.e., which quality of care and long-term outcomes can be achieved at given prices). In his 2010 paper on value-based health care (VBHC), Porter observed that “Achieving high value for patients must become the overarching goal of health care delivery, with value defined as the health outcomes achieved per dollar spent. This goal is what matters for patients and unites the interests of all actors in the system” [1]. Therefore, to define value, it is important to define relevant outcomes (including toxicity/complications) on one hand and costs (including waste and unnecessary expense) on the other. VBHC aims to provide outcomes favorable to patients at the fairest possible costs. It should keep health systems sustainable and continually improving. In an era of “pay for performance,” a pro-active role of the health care professionals in defining quality is increasingly important [2].

This review presents a summary of some important aspects of quality of care in HNC. Because financial costs in health care are notoriously difficult to measure and much more dependent on particular features of individual systems, the focus will be indicators of quality and outcome in HNC care, how to measure them, and how to use them to improve care.

### Quality Indicators and Registries

Within healthcare, there is growing recognition of the need for accurate and germane information to improve quality because “Rigorous, disciplined measurement and improvement of value is the best way to drive system progress” [1]. Hence, the acquisition of data defining the indicators should be incorporated in quality registries, which should be prospective, comprehensive, and comparable. The virtues of measuring the quality of medical care and, thereby, fostering its continuing improvement have been recognized for many years [3].

In 2006, Porter and Teisberg’s book, “Redefining Health Care” on VBHC [4], resulted in the founding of the International Consortium for Health Outcomes Measurement (ICHOM) (http://www.ichom.org/). ICHOM has subsequently developed minimally acceptable data sets for several diseases, including colorectal, lung, and breast cancer [5–7]. Other organizations have developed additional data sets, including for HNC [8•]. To establish the relevant indicators of quality, suitable indices should ideally be defined by multidisciplinary groups, using approaches such as the RAND-modified Delphi method and by engaging all stakeholders or actors, including patients [8•]. Other means to establish registries to improve quality of care in daily practice warrant consideration; quality assurance programs in the context of clinical trials could be elaborated to serve as the basis for prospective registries as well [9].

Quality registries offer several routes to improve health care. These include the opportunity to monitor quality of care delivered and to provide feedback when necessary. They also make possible auditing to identify differences in patterns of care, potentially pointing toward improvement. They offer the opportunity to conduct “outcomes research,” to study “real world” results [10]. In contrast to clinical trials, which include only subjects meeting defined criteria and interested practitioners or centers, the unselected population can be analyzed through sufficiently comprehensive registries. However, challenges reside in the burden of gathering relevant underlying data, creating the necessary infrastructure, and addressing data ownership and privacy concerns.

The early adoption, decades ago, of VBHC principles in Sweden has already resulted in the establishment of more than 100 government-funded registries pursuing the systematic collection of outcomes, which can be used to guide patient choice and support learning and quality improvement [11]. The Swedish Head and Neck Cancer Register (SweHNCR), functional since 2008, is an already well-established HNC quality registry (http://kvalitetsregister.se/englishpages/findaregistry/registerarkivenglish/nationalqualityregistryforheadandneckcancer.2287.html). Additional examples of how these can provide information to improve quality of care are emerging in the HNC arena [8, 12, 13]. In low resource settings similar initiatives may be difficult to implement but ideas on how to proceed in less developed settings have been published [14].

Quality indices can include structure, process, and outcome indicators [15] (Table 1). Which of these are most important is subject to debate. Optimizing processes of care may be imperfect surrogates for improvement rather than truly increasing the actual quality and value derived by the patient. While outcomes and costs are the “real” determinants of value, assessing appropriate process indicators may influence eventual outcomes and provide tools to improve them.

**Table 1:** Quality indicators

	Structure	Process	Outcome
Health care professionals’ perspective	Volume, centralization, registry	MDT, adherence to guidelines, waiting times, network	Survival, adverse events
Patients’ perspective			Quality of life (PROs), functional outcome, experiences (PREs)

### Structure Indicators

Structure indicators reflect the environment of health care delivery and provide the basis to provide good care. They consist of two major factors: personnel and equipment. In addition to the number of specific caregivers and the diagnostic and therapeutic facilities, the multidisciplinary network (with its degree of centralization) also belongs to the structure indicators. The medical record and IT support may be considered in this category as well. Structure indicators may be relatively easy to define and to measure. However, they alone do not guarantee quality and may not represent (good) surrogates for something that the patient actually receives.

The impact of structure might be reflected, for example, in publications on survival rates of HNC in European countries [16–18], where clear differences are demonstrated. Although the reasons for these differences are uncertain, one factor may be the organization and centralization of care. The Nordic countries, UK, Ireland, and the Netherlands, with traditions of decades of centralization and multidisciplinary teams and clinics, seem to have better survival outcomes [16–18], and many initiatives are aiming at centralization with the objective to increase quality and reduce costs [19]. However, demographics may differ considerably, resulting in difficulties of comparison between countries.

Centralization is an important feature of structure and closely related to both volume and experience rate. By centralization with higher volumes, the experience of the involved health care professionals increases and economies of scale also permit cost-efficient investments, in (highly) specialized personnel, infrastructure, and equipment. Analyzing the impact of centralized care of rare malignancies, including rare HNCs in Europe [20] confirms the favorable effects of centralization on survival in rare tumors. Although the benefits of centralization of care of rare diseases are now widely accepted, they typically reflect a long process. Very strong scientific evidence is needed to drive physicians to alter their patterns of care [21].

Volume is sometimes also cited as an outcome measure, which can be confusing. The differences between low and high volumes are not well defined, and the definition of volume is not simple. Systematic review of systematic reviews establishes a relationship between volume and outcome [22]. However, the definition of minimum required case volumes remains unanswered for most situations.

Many studies demonstrate a (positive) correlation between higher volume and better outcomes including those for HNC [23–31] and salivary gland cancer [32]. One example is the relation between hospital case volume and outcomes of laryngectomy. Reduced mortality, morbidity, length of hospitalization, and costs were associated with higher hospital volume [24]. Another example in HNC is the recently published association between involved surgical margin rates and facility volume for patients with oropharyngeal carcinoma treated by transoral robotic surgery [33]. High-volume centers have significantly fewer positive surgical margins in oncological surgery than low-volume facilities. However, this easily measured indicator should be considered a surrogate endpoint.

In addition to the demonstrable advantages of centralization, other aspects merit consideration. Patient traveling distances are increased, potentially reducing adherence, patient satisfaction, and quality of life [34].

It may be that HNC cases have variable complexity and the virtues of centralization of oncologic treatment with low complexity should be balanced against the associated costs of treatment of these cases in tertiary referral centers. However, even in less complex cases experience is important for optimal outcomes and appropriate costs. In this regard, organization of the HNC patient’s pathway into a “hub and spoke” network model should improve patient satisfaction and quality of care, as suggested with other cancers [35, 36].

### Process Indicators

Process indicators are meant to measure important steps in the healthcare delivery pathway. The process includes all steps of the treatment trajectory, from appreciation of the lesion through diagnostic procedures, treatment, follow-up, and survivorship. Due to its complexity and impact in cancer care, the decision-making process is often separately described but still also as a part of the treatment trajectory.

#### Multidisciplinary Teams, Integrated Care

One simple process indicator is the presence of a multidisciplinary team (MDT).

HNC, perhaps more than any other tumor, requires a multidisciplinary approach. The optimal collaboration and concertation of all involved disciplines, both medical and allied health professions, is an important basis for quality of care [37]. The importance of MDTs and their impact on outcomes is increasingly investigated and recognized [38–41]. Their implementation and optimization have been submitted to study [39, 42, 43]. MDTs have been associated with improved efficiency and completeness of care [44]. Enthusiasm for adoption of MDTs varies between countries. Some have had them for decades, and some health systems are still attempting to establish them.

The exact definition of what should constitute a MDT varies, but head and neck oncology MDTs are typically composed of the three major disciplines involved in the treatment of these malignancies: radiation oncology, medical oncology, and head and neck surgery (including reconstructive surgery). In addition, specialists such as radiologists, pathologists, geriatricians, dentists, and allied health professionals such as nutritionists, cancer nurses, physiotherapists, oral hygienists, social workers, and psychologists are key participants. Typically, these care-givers are mainly involved in the decision-making process during MDT meetings; however, specific patient or tumor characteristics regularly necessitate the involvement of other disciplines.

Establishment of a “MDT” as such does not guarantee uniformity of decisions, and there are no standardized methods to conduct MDT meetings. Guideline adherence may vary, and decisions often vary depending on preferences of individual professionals attending the MDT; they may be influenced by factors such as authority, institutional preferences, or school. For optimal functioning of multidisciplinary teams collegiality or a good “team climate” is important [45].

Rankin et al. conducted a descriptive study on team functioning, the role of team meetings, and evidence use in MDTs. They found that MDT treatment decisions were based on group consensus (92%), adherence to clinical practice guidelines (57%), or other evidence-based medicine sources (33%) [46]. Kirkegård et al. showed a high heterogeneity in the staging of pancreatic tumors and in the definition of resectability between European MDTs [47]. Similar variability is to be expected between head and neck MDTs.

As suggested by Guy et al., MDT meetings are necessary at every stage of disease [43].

Although there are currently no doubts about the benefits of MDTs, introduction and quality assurance are challenging, particularly in low-resource settings. Difficulties in establishing an MDT have also been identified: it is time-consuming, synchronizing physician availability may be challenging, and sometimes there are concerns that waiting for the MDT meeting will engender treatment delay [48]. In order to answer these issues, teleconsultation services could help, particularly for specific questions, such as pathology review or speech pathology consultation [49].

#### Adherence to Guidelines

Guidelines form the basis to standardize and optimize delivered care. They are usually developed according to evidence-based principles and use the best information available. They are considered superior to physician experience-based treatment practice. Therefore, adherence to guidelines is considered a process indicator. However, application of guideline recommendations to a specific case should be undertaken with care. Patients in practice often significantly differ from the population of the trials, which often serve as a basis for the guideline.

Prospective registries/databases used for quality purposes may also serve as sources for outcomes research and guidelines. In contrast to randomized trials in which the population is selected by inclusion criteria, data generated by quality or disease registries offer the opportunity to study outcomes in clinical reality and may form the basis for guideline development and maintenance.

The development and maintenance of head and neck oncology guidelines should, of course, be multidisciplinary, but the involvement of patients in the development of guidelines and in research is increasingly acknowledged as important and valuable [50–52]. However, it is likely to be difficult to demonstrate that their inclusion is associated with improved outcomes [53].

Although the quantity and quality of information provided by guidelines is accumulating, actual adherence to recommendations involves barriers [54] and keeping the guidelines up to date is challenging. An example is the adherence to thyroid cancer management guidelines and application of the guideline of timely inception of postoperative radiation therapy, which has room for improvement [55, 56].

#### Time to Treatment Initiation

Another prominent process indicator is the time to treatment initiation.

As HNC is often relatively fast growing in an anatomically complex and functionally vulnerable site, it is conceivable that any tumor progressions would affect survival and/or quality of life.

The detrimental effect of prolonged time to treatment initiation intervals on outcome is difficult to study but both oncological outcomes (survival) and/or functional (quality of life) and psychological (e.g., anxiety) disadvantages are to be expected [57•, 58–60]. Conversely, outcome is influenced by many factors and it is difficult to demonstrate the effect of one single factor. Anyway, tumor progression may necessitate intensified treatment, compensating the potential loss of survival but increasing toxicity and costs (which are not often measured but are important in the equation of value of care).

Due to the complexity of HNC care, often requiring multiple imaging techniques, sometimes diagnostic endoscopy under general anesthesia, and often involving patients with multiple medical problems/comorbidities, timely start of treatment can be challenging but is to be desired [61]. An increase in time to treatment initiation in the USA is reported In recent publications, including its effect on survival [57•, 62, 63]. In Denmark this topic has been studied and a similar trend reported. The findings resulted in nation-wide initiatives to expedite the diagnostic maneuvers and initiation of treatment [64, 65•]. Similar guidelines or targets have been defined in other countries as well. In 2005, the UK National Institute for Health and Care Excellence (NICE) first published clinical guidelines for the recognition and referral of suspected cancer. Updated in 2015, the Department of Health currently has specified times within which patients with suspected cancer should be seen; the national target is 14 days from the day of referral from primary care [66, 67].

#### Networks

Another relevant aspect may be the organization of care in a network for communication and collaboration between secondary and tertiary referral centers [68]. Timely referral and execution of diagnostic procedures may reduce time to treatment initiation and therefore enhance outcomes. Doing the proper diagnostic procedures according to the correct protocols and guidelines in dedicated head and neck centers (eliminating need to repeat them) and avoiding unnecessary procedures adds to cost-effectiveness and timely treatment. Communication and sharing of imaging are facilitated by the possibilities of modern technology such as teleconferencing to organize consultation. Larger networks of head and neck oncologic care can pursue clinical research to increase knowledge of rare diseases [69].

### Outcome Indicators

A broad spectrum of both objective and subjective measures comprise outcome indicators. Traditionally, survival has been considered the most important outcome indicator in cancer care, but it is now recognized that adverse events and quality of life are also important. Prioritizing these outcomes may significantly differ between patients, their caregivers, and members of care teams [70••].

Quality of life measured by patient reported outcomes (PROs) and experiences (PREs) is subjective by nature. This concerns both disease-specific and generic health-related quality of life measures. The comparison of these indicators between different patient cohorts is difficult because outcome measures are dependent on external factors. Priorities may change. For instance, elderly HNC patients favoring quality of life to prolonged life [71, 72]. Hence, the introduction of geriatric screening may lead to shorter survival; however, patient satisfaction (expressed in PREs and PROs) may increase [73].

#### Survival

As mentioned above, survival has been regarded traditionally the most important outcome parameter. Survival rates have been improved through the years (https://seer.cancer.gov/csr/1975_2016/browse_csr.php?sectionSEL=2&pageSEL=sect_02_table.08). However, it is uncertain to which extent progress in both the surgical (e.g., robotic surgery) and non-surgical treatment modalities (e.g., proton therapy and immunotherapy) during the past decade have added to survival benefit.

Introduction of new treatment options or paradigms as such is not always accompanied by better survival but may instead be primarily driven by less associated toxicity or morbidity of a treatment in combination with unaltered survival. An example is the introduction of organ preservation treatment regimens [74, 75].

#### Quality of Life and Functional Outcomes

Quality of life and of supportive care have been increasingly recognized critical to delivering optimal results [76, 77•]. They are closely related to functional outcomes of treatment such as speech articulation, swallowing, taste, jaw opening, and saliva production in head and neck oncology. However, there are many important domains for head and neck cancer patients, and assessing the impact of treatment is demanding [78].

The prospective collection of these data provides important information on “real life” care but is challenging for both PROs and functional outcomes [79••, 80]. The acquisition of PROs through questionnaires requires motivating patients to complete them. Even in the context of clinical trials, the compliance of patients in providing the information may be poor unless the individual results are also actually used in practice [81]. Nonetheless, for (shared) decision making and weighing oncological outcomes against quality of life outcomes, the measurement and knowledge of quality of life issues are essential [82–86].

#### Patient Experiences

Another parameter reflecting quality of care is how patients experience its delivery. It is important to know the needs and preferences of patients and assess their perceptions and experiences [87, 88]. There are several methods to measure these preferences and experiences including interviews and the use of patient-reported experience (PRE) questionnaires.

#### Adverse Events (Complications and Toxicity)

Adverse event (AE) is a term including harmful events such as postoperative complications and toxicity. AEs have always been a part of health care. However, their corresponding systematic measurement has a short history. The US National Cancer Institute has developed a system (Common Terminology Criteria for Adverse Events—CTCAE) to allow health-care providers to score and to compare AEs. Treatment-related AEs form the 14th most common cause of disease burden worldwide with significant financial consequences [89]. The most common reported AEs are health-care-associated infections (or hospital-acquired infections) [90]. In head and neck oncology, treatment-related AEs are well-defined and sophisticated efforts and strategies have been developed to limit them. Radiation-induced toxicity significantly affects patients’ short- and long-term quality of life. Operations also engender unwanted sequelae and complications with related mortality and morbidity. Prediction models have been developed, which may help in the decision-making process and in choosing the best treatment [91]. Quality assurance in head and neck surgery suggests structured registries including those of surgical complications and their risk factors [92]. In specific patient populations (e.g., elderly or infirm) adverse events have other predictors, other consequences, and sequelae [93]; it is uncertain how these should influence therapeutic decision making [94].

In patients with recurrent and/or metastatic disease, the application of systemic treatments in the last month of life should be considered another care quality indicator. Treatment in these patients may cause a high adverse event burden and a consequent deterioration in already poor quality of life. For this reason, there is a general recommendation not to administer chemotherapy near the end of life [95].

## Nomograms, Prognostic Models, and Decision Models

“Big data” may provide valuable information and form the basis for nomograms and decision models that are receiving growing attention in health care. These tools may allow prediction of “individual” outcomes, which contributes to both physician and patient decision making and perhaps also to better outcomes. Prediction models have been significantly improved in the last decades with setting up large databases and with the development of analytical tools and methods. Using “big data,” prediction models became more reliable. Examples of prognostic models combining patient characteristics, such as age, sex, and comorbidities and also tumor factors, primary site, and TNM classification have been published [96] including their validation on other populations [97]. However, the issues of application to different populations have not been entirely resolved [98].

Models for specific patient categories [99] or clinical questions (e.g., elective neck treatment, post-operative radiation) have been published [100]. These may eventually be integrated into the decision-making process in order to enhance survival, quality of life, adverse event outcomes, and reduce health care costs [101].

## Learning from Variation

Even within the context of clinical trials and even between comparable centers, outcomes may vary. Variation in adherence to guidelines and quality of delivered care has been documented within the context of randomized trials in which quality assurance is included and also in studies using data from population/cancer registries [31, 102, 103, 104••]. Analysis of these findings may lead to improved quality and outcomes by identifying best practices and learning from them. Translating these analyses to recommendations and guidelines should favorably influence outcome results in clinical practice [105, 106].

## Final Remarks and Considerations

In this paper focus has been put on quality of care but, with increasing costs of health care in particular, the question of costs is relevant as well. However, as already stated in the introduction, financial costs in health care are notoriously difficult to measure and much more dependent on particular features of individual systems. As, in the concept of VBHC, value is defined as outcomes relative to costs, there is also risk: focusing on cost reduction to increase “value.” As Porter stated, “cost reduction without regard to the outcomes achieved is dangerous and self-defeating, leading to false “savings” and potentially limiting effective care” [1]. Therefore, it is crucial for health care professionals together with their patients to properly define quality and relevant outcomes.

Clearly, there are many ways to define and improve the quality of delivered care and to create value for patients requires knowing what really matters to them (their needs and preferences). Moreover, the creation of value is a multifaceted and much more than “What do I get for this price?” How, where, when, and by whom is value created? It may be worthwhile to look for parallels in other sectors. Heinonen et al. have assessed the multi-dimensional aspects of value creation in the online-commercial world by unraveling it into matrices. They found that firms tend to miss values because they focus on their own resources and capabilities, rather than expanding their scope (https://hbr.org/product/strategies-for-creating-value-through-individual-and-collective-customer-experiences/BH956?sku=BH956-PDF-ENG). Does this translate into health care and if so, how?

Next to defining quality and outcomes reflecting this, it is essential to collect the relevant “real world” data. Only this will allow both analysis of the contributing factors and potential novel parameters to improve patient outcomes. Establishing quality registries is a way to acquire data showing where quality improvements can be achieved and optimizing the balance between outcomes and costs. Emerging “big data” from these registries can also be used to develop tools to help to provide individualized and tailored treatments, thus improving individual patient outcomes and adding to the overall improvement of outcomes for the entire population. To be useful and feasible, these registries should be prospective, comprehensive, and comparable and registration burden should be minimized. Moreover, a system is needed to translate these outcomes into actions to actually improve, to remeasure, and in such a way to create a quality cycle.
